# Kisspeptin Regulation of Genes Involved in Cell Invasion and Angiogenesis in First Trimester Human Trophoblast Cells

**DOI:** 10.1371/journal.pone.0099680

**Published:** 2014-06-12

**Authors:** Víctor A. Francis, Aron B. Abera, Mushi Matjila, Robert P. Millar, Arieh A. Katz

**Affiliations:** 1 MRC/UCT Receptor Biology Unit, Institute for Infectious Disease and Molecular Medicine and Division of Medical Biochemistry, Faculty of Health Sciences, University of Cape Town, Cape Town, South Africa; 2 Department of Obstetrics and Gynaecology, Groote Schuur Hospital, Faculty of Health Sciences, University of Cape Town, Cape Town, South Africa; 3 Mammal Research Institute, Zoology and Entomology, University of Pretoria, Pretoria, South Africa; 4 Centre for Integrated Physiology, University of Edinburgh, Edinburgh, United Kingdom; Swedish Medical Center, United States of America

## Abstract

The precise regulation of extravillous trophoblast invasion of the uterine wall is a key process in successful pregnancies. Kisspeptin (KP) has been shown to inhibit cancer cell metastasis and placental trophoblast cell migration. In this study primary cultures of first trimester human trophoblast cells have been utilized in order to study the regulation of invasion and angiogenesis-related genes by KP. Trophoblast cells were isolated from first trimester placenta and their identity was confirmed by immunostaining for cytokeratin-7. Real-time quantitative RT-PCR demonstrated that primary trophoblast cells express higher levels of GPR54 (KP receptor) and KP mRNA than the trophoblast cell line HTR8Svneo. Furthermore, trophoblast cells also expressed higher GPR54 and KP protein levels. Treating primary trophoblast cells with KP induced ERK1/2 phosphorylation, while co-treating the cells with a KP antagonist almost completely blocked the activation of ERK1/2 and demonstrated that KP through its cognate GPR54 receptor can activate ERK1/2 in trophoblast cells. KP reduced the migratory capability of trophoblast cells in a scratch-migration assay. Real-time quantitative RT-PCR demonstrated that KP treatment reduced the expression of matrix metalloproteinase 1, 2, 3, 7, 9, 10, 14 and VEGF-A, and increased the expression of tissue inhibitors of metalloproteinases 1 and 3. These results suggest that KP can inhibit first trimester trophoblast cells invasion via inhibition of cell migration and down regulation of the metalloproteinase system and VEGF-A.

## Introduction

Extravillous trophoblast (EVT) invasion of the maternal uterine wall is a prerequisite for successful placentation and healthy pregnancy. During the first trimester of pregnancy, EVTs invade the maternal decidua and the myometrium, remodelling the spiral arteries to ensure an appropriate nutrient and gas exchange between the fetus and the mother [1].

The dysregulation of this process has been shown to cause complications during pregnancy. Poor trophoblast invasion is associated with preeclampsia and intrauterine growth restriction (IUGR) [2], [3], whereas an excessive invasion leads to placenta accreta or percreta [4].

Trophoblast invasion closely resembles tumour metastasis [5], as trophoblast cells utilize the same molecular mechanisms as cancer cells for their migratory and invasive functions. Among these mechanisms, the matrix metalloproteinase (MMP) system is of great importance. MMP2 and MMP9 have been shown to play an important role in EVT invasion [6], [7]. The activity of these MMPs can be further regulated by their counterparts, the tissue inhibitors of metalloproteinases (TIMPs) [8].

Despite this resemblance to metastasis, EVT invasion is tightly regulated. Several factors are involved in ensuring correct invasion. For example, transforming growth factor-β1 (TGF-β1) is produced by first trimester decidual cells and limits trophoblast invasion by stimulating TIMP expression [9]. Moreover, TNFα, produced by decidual macrophages, limits trophoblast invasion through elevation of plasminogen activator inhibitor-1 (PAI-1) [10]. Kisspeptins (KPs) have also been identified as regulators of trophoblast invasion in first trimester human trophoblast cells [11] and in the immortalized trophoblast cell line HTR8SVneo (HTR8) [7].

KPs peptides are derived from the KISS-1 gene [12] that encodes a 145 amino-acid polypeptide [13], that is proteolytically processed to kisspeptins of 54, 14, 13 and 10 amino acids [13]–[15]. KP-54 (metastin) was first described as an antimetastatic molecule [13], [14]. A role for KP has also been described in regulating puberty onset via its regulation of gonadotropin-releasing hormone (GnRH) secretion [16].

KP binds to the G-protein coupled receptor GPR54, also known as AXOR12 and KISS-1R. Together they are able to activate phospholipase C_β_ (PLC_β_) possibly via G_q/11_ and resulting in increased intracellular Ca^2+^ [15]. KP has also been shown to activate ERK signalling pathway in GPR54-transfected CHO cells and in the immortalized trophoblast cell line HTR8SVneo [7], [14].

Both KP and GPR54 transcripts peak in expression in the human placenta during the first trimester of pregnancy and thereafter their expression decreases [17], while serum KP levels of pregnant women increase during pregnancy until term [18]. Bilban et al. [11] showed that GPR54 is expressed in syncitiotrophoblast, villous cytotrophoblasts and extravillous (evCT) cytotrophoblasts, while KP is restricted to the syncytiotrophoblast. However, recently, Park et al. reported that GPR54 is expressed only in syncitiotrophoblast and not in cytotrophoblasts, whereas KP was most abundantly expressed in syncytiotrophoblasts and moderately in cytotrophoblast [19]. Cartwright et al. detected KP and GPR54 mainly in syncitiotrophoblast, and at a lower level also in the villous cytotrophoblast [20]. While, a recent study using immunohistochemistry, detected expression of kisspeptin and GPR54 in syncytiotrophoblasts and cytotrophoblasts [21].

Despite the initial evidence gathered by Bilban et al. demonstrating the KP inhibits migration of trophoblast cells in explants and primary cultures [11], no studies have targeted the effects of KP on the regulation of genes involved in cell invasion and angiogenesis in trophoblast primary cultures. We have therefore established primary cultures of first trimester human trophoblast cells and studied the effect of KP treatment on trophoblast migration and expression of genes involved in remodelling of the extracellular matrix and angiogenesis.

## Materials and Methods

### Reagents and antibodies

Kisspeptin-10 (KP) and its antagonist (p356) were custom-synthesized by EZ Biolabs. p356 is a KP antagonist derived from antagonist p234 [22]. The source of all other reagents was Sigma unless otherwise indicated.

Antibodies used for Western blot were rabbit anti-GPR54 (GTX100374, GeneTex), rabbit anti-Kisspeptin (ab80994, Abcam), rabbit anti-β-actin (sc-1616, Santa Cruz), rabbit anti-ERK1/2 and rabbit anti-P-ERK1/2 (9102 and 9106, Cell Signalling). HRP-conjugated anti-mouse and anti-rabbit (Santa Cruz) were used as secondary antibodies. Antibodies used for immunocytochemistry were rabbit anti-GPR54 (R2 1212 serum, EZ Biolabs), mouse anti-cytokeratin-7 (M7018, Dako), mouse anti-vimentin (M0725, Dako), rabbit IgG anti-human KSHV GPCR (Cell Sciences) and mouse IgG HA-Probe (F-7) (sc-7392, Santa Cruz). Cy3-conjugated anti-mouse and Alexa 488-conjugated anti-rabbit (Jackson Immuno Research) were used as secondary antibodies.

### Tissue collection

Placenta of first trimester were obtained from elective terminations of pregnancy at Groote Schuur Hospital with approval of the Human Research Ethics Committee of the University of Cape Town who approved the study including the patient consent procedure (REC REF: 080/2008). Only patients who signed the patient's consent form were recruited to the study and all signed patient consent forms have been filed and kept. Tissues were collected, washed with ice-cold PBS and processed immediately.

### Trophoblast isolation and cell culture

Placenti were processed to obtain trophoblast primary cultures as described by Wu et al [23]. Briefly, placental tissue was cut into small pieces and digested four times for 30 min with 0.25% trypsin and DNAse I (300 U/ml). Loose cells were then separated by centrifugation using a Percoll gradient column (Sigma). The isolated trophoblast cells were plated in dishes and incubated for 40 min to allow macrophages to adhere to the plates. The nonadherent trophoblast cells were transferred to fresh plates and cultured at 37°C in 10% CO_2_ in RPMI 1640 medium (Gibco - Invitrogen) containing 2 mM L-Glutamine and supplemented with 10% FCS. and 1% Penicillin/Streptomycin. HTR8SVneo extravillous trophoblast-derived cells were obtained from Dr. Charles H. Graham [24] and were maintained in the same culture conditions as primary trophoblast cells.

### Immunocytochemistry

Cells were fixed on coverslips or on 48 well plates and permeabilized by a 10 min methanol treatment at −20°C. Cells were stained with mouse anti-cytokeratin-7 (1∶50 dilution), rabbit anti-GPR54 (1∶50), mouse anti-vimentin (1∶50), mouse IgG HA-Probe (F-7) (1∶50) or rabbit IgG anti-human KSHV GPCR (1∶50). Thereafter, cells were stained with the secondary antibodies, Cy3-conjugated anti-mouse (1∶500) or Alexa 488 conjugated anti-rabbit (1∶500). Cell nuclei were stained with DAPI (1∶2000).

### RNA extraction, quantitative real-time RT-PCR (qPCR) and TaqMan Gene Array

RNA was extracted using Trizol (Invitrogen) following the manufacturer's instructions. One microgram of each mRNA sample was used for synthesis of first-strand cDNA using the MultiScribeRT enzyme (Applied Biosystems). qPCR amplification (40 cycles of 15 sec at 95°C and 1 min at 60°C) was performed using the Bioline SensiMix II reagent (Celtic Diagnostics) in a CFX96 qPCR machine (BioRad). The housekeeping gene cyclophilin A (CYPA) was used for normalization. The names of genes, their accession number and primers used are indicated in Table 1. The TaqMan array for Human Extracellular Matrix & Adhesion Molecules (4414133, Life Technologies) was performed using the same qPCR machine and conditions described above.

**Table 1:** Genes and primer sequences used in quantitative real-time PCR.

Gene Name	Accession Number	Forward Primer (5′ to 3′)	Reverse primer (5′ to 3′)
Cyclophylin A	NM_021130.3	CCCACCGTGTTCTTCGACAT	CCAGTGCTCAGAGCACGAAA
Kisspeptin	NM_002256.3	CTCACTGGTTTCTTGGCAGCTAC	TTCTAGCTGCTGGCCTGTG
Kisspeptin receptor (GPR54)	NM_032551.4	GCTGGTACGTGACGGTGTTC	AGAGCCTACCCAGATGCTGAG
Matrix metalloproteinase 1	NM_002421.3	TCCCTAGAACTGTGAAGCATATCG	GCCAATTCCAGGAAAGTCATGTG
Matrix metalloproteinase 2	NM_004530.4	GGCGGTCACAGCTACTTCTTC	AGCCTAGCCAGTCGGATTTG
Matrix metalloproteinase 3	NM_002422.3	GGTACGAGCTGGATACCCAAGAG	GCTATTTGCTTGGGAAAGCCTGG
Matrix metalloproteinase 7	NM_002423.3	CAAAGTGGTCACCTACAGGATCG	TCCTCGCGCAAAGCCAATC
Matrix metalloproteinase 9	NM_004994.2	TGGAGGTTCGACGTGAAGG	AAATAGGCTTTCTCTCGGTACTGG
Matrix metalloproteinase 10	NM_002425.2	GAGAAAGCTCTGAAAGTCTGGGAAG	TCCAGGTGGGTAGGCATGAG
Matrix metalloproteinase 14	NM_004995.2	TCCAGGGTCTCAAATGGCAAC	TTGCGAATGGCCTCGTATGTG
Matrix metalloproteinase 16	NM_005941.4	AGAATGTCAGTGCTGCGCTC	ACCTCTTGTCTGGTCAGGTACAC
TIMP-1	NM_003254.2	GGCTTCACCAAGACCTACACTG	GGTCCGTCCACAAGCAATGAG
TIMP -2	NM_003255.4	CACCCAGAAGAAGAGCCTGAAC	CTGTGACCCAGTCCATCCAGAG
TIMP -3	NM_000362.4	CCGAGGCTTCACCAAGATGC	ATCTTGCCATCATAGACGCGAC
VEGF-A	NM_001171623.1	ACATCTTCAAGCCATCCTGTGTG	CTCTCCTATGTGCTGGCCTTG
Angiopoietin-like 4	NM_015985.2	TCTCCGTACCCTTCTCCACTTG	TGGCCGTTGAGGTTGGAATG

### Protein extraction and SDS-PAGE

After stimulation, cell monolayers were placed on ice, washed with ice-cold PBS and lysed in solubilisation buffer (150 mM NaCl, 1% Nonidet-P40, 1 mM EDTA pH 8.0) supplemented with 1 mM sodium orthovanadate, 5 mM sodium pyrophosphate, 50 mM sodium fluoride, and Complete EDTA-free protease inhibitor and PhosSTOP phosphatase inhibitor cocktail tablets (Roche). Cell lysates were clarified by centrifugation at 14,000 rpm for 10 min. Thereafter, a 100 µl of clarified cell lysate was mixed with an equal amount of Laemmli sample buffer and resolved by SDS-PAGE at 120 V for 1.5 h.

### Immunoblotting

After electrophoretic separation by SDS-PAGE, proteins were transferred on to a Hybond-P PVDF membrane (Amersham - GE Healthcare). PVDF membranes were incubated in blocking solution (5% milk, 50 mM Tris HCl pH 7.0, 0.05% Tween-20) for 1 h and probed overnight with mouse Anti-GPR54 (1∶500 dilution), rabbit Anti-Kisspeptin (1∶100 dilution), rabbit Anti-ERK1/2 and Anti-P-ERK1/2 (1∶1000 dilution). Then Anti-Mouse or Anti-Rabbit HRP-conjugated secondary antibodies (1∶5000 dilution) were added followed by addition of SuperSignal West Pico chemiluminescent substrate (Thermo Scientific) and quantified using the BioSpectrum 500 Imaging System (UVP).

### Scratch migration assay

Trophoblast cells were grown to confluence on 12 or 24-well tissue culture plates. A scratch was created with a pipette tip and cells were then washed three times with PBS at 37°C to remove any loose cells. Trophoblast cells were either not treated or treated with p356 only or KP or a combination of both KP and its antagonist (p356). Cells were then incubated in serum free media for 48 h at 37°C with 10% CO_2_. Cell scratches were photographed at 0 h and 48 h after treatment, with the width of the scratch recorded at each time using the Zeiss Axiovert 200 M fluorescence microscope (Zeiss). Migration was evaluated by measuring the distance between scraped edge on both sides at 0 h and measuring the distance between the furthest migrated cells at 48 h for the indicated treatments. Migration is expressed as relative migration which is a ratio between the distance the cells have migrated after 48 h to the distance between scraped edge on both sides at 0 h for each treatment. The migration of cells with the treatments was expressed relative to the migration of cells in absence of any treatment which was considered as 1.

### Statistical analyses

All analyses were performed using GraphPad Prism 5.0. A two-way ANOVA followed by Tukey multi comparison post-test was performed. Statistical significance was set at p<0.05 and experiments were repeated 3–5 times.

## Results

### Expression and activity of KP and GPR54 in primary trophoblast cells

**Figure 1:**
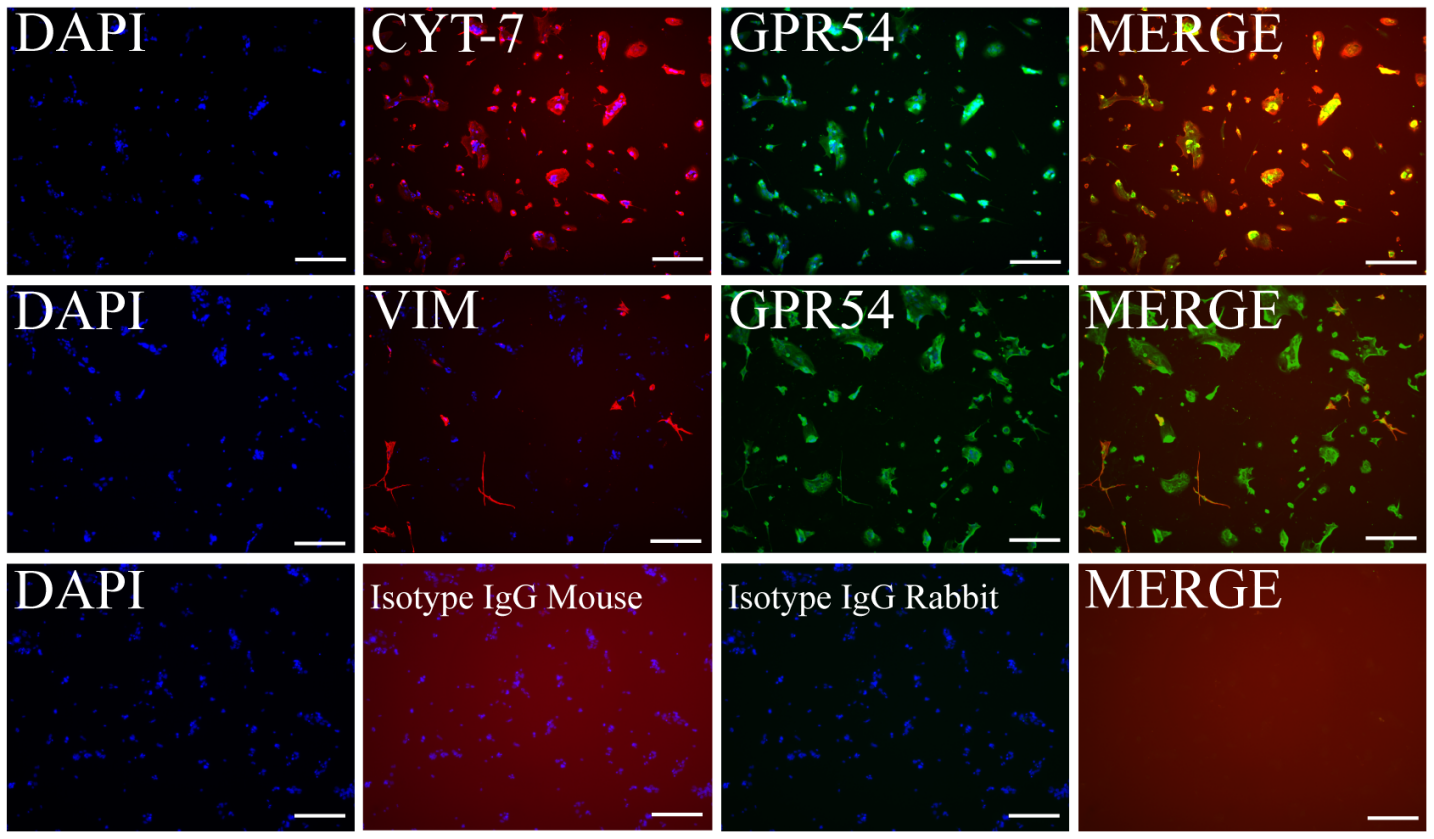
Characterization of primary cultures of first trimester trophoblast cells. Staining of primary cultures of isolated first trimester trophoblast cells. Top row, **s**taining with DAPI (blue) which labels cell nuclei, anti-cytokeratin-7 (CYT-7, red), anti-GPR54 (green) and the merge of staining with anti-cytokeratin-7 (red) and anti-GPR54 (green). Middle row, **s**taining with DAPI (blue), anti-vimentin (VIM, red), anti-GPR54 (green) and the merge of staining with anti-vimentin (red) and anti-GPR54 (green). Bottom row, staining with DAPI (blue), isotype matched mouse and rabbit IgG as negative controls and their merge. Scale bar indicates 200 µm.

In order to determine the fraction of trophoblast cells in the primary cultures, cells were stained with anti-cytokeratin-7, which is a positive marker for trophoblast cells and with anti-vimentin which is a marker of mesenchymal cells. Figure 1 shows a representative image of cytokeratin-7, vimentin, GPR54 and DAPI nuclei staining. By counting the number of cells positive for cytokeratin-7 or vimentin staining relative to total cells in each stained preparation (n = 9), it was determined that the percentage of cytokeratin-7 positive cells (top row) was 88.2 ± 1.6%, while the percentage of vimentin postive (middle row) was 10.4% ± 1.8%. Staining with isotype matched IgG as control (bottom row) did not show any staining demonstrating the specificity of the staining with anti-cytokeratin-7, anti-vimentin and anti-GPR54. Staining with anti-GPR54 (top and middle rows) shows that essentially all the cells express GPR54. Furthermore, the merge demonstrates that cells expressing cytokeratin-7 or vimentin also express GPR54. These results together demonstrate that about 90% of the cultured cells are trophoblast cells that co-express cytokeratin-7 and GPR54.

**Figure 2:**
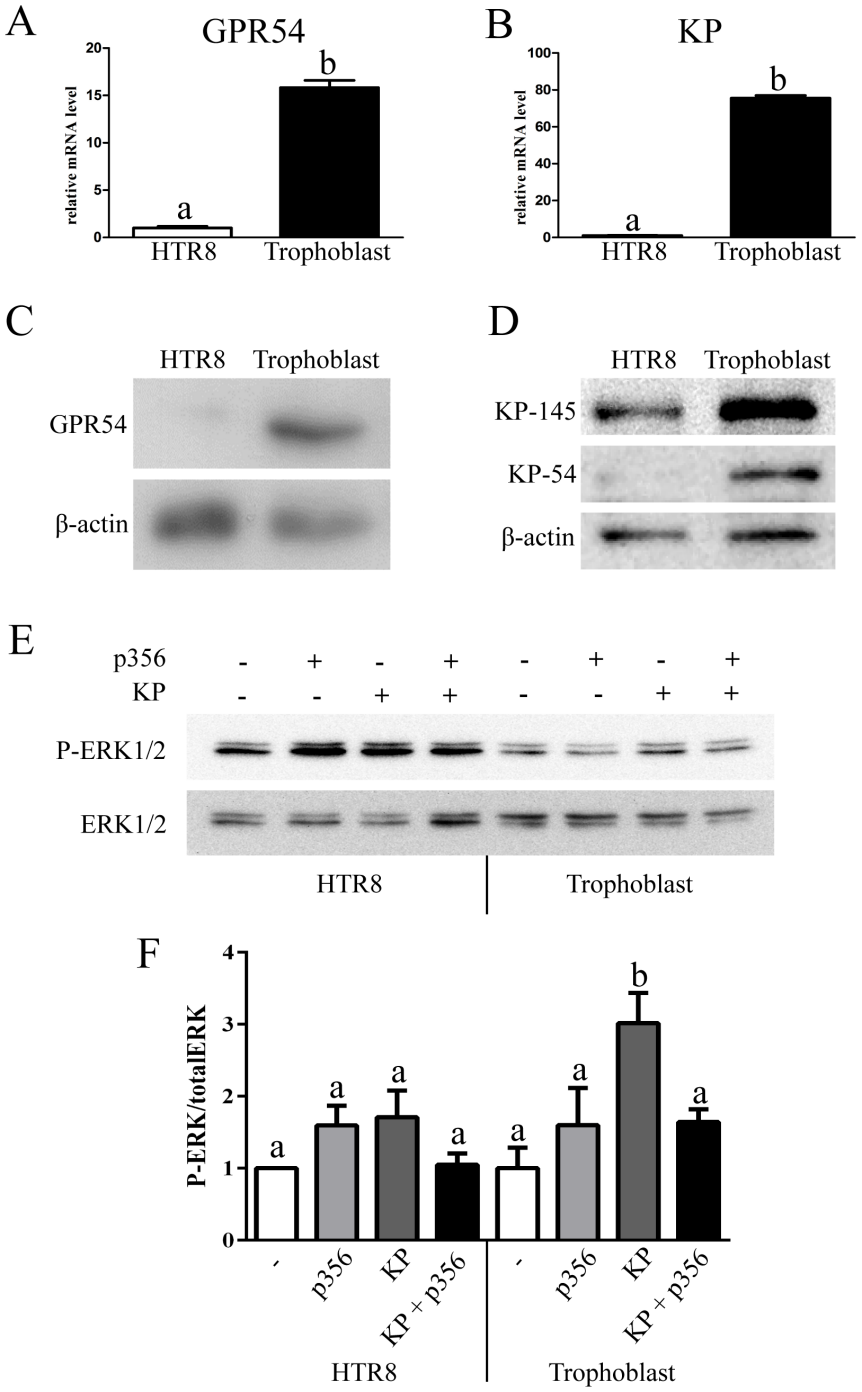
Expression of GPR54 and KP and KP induction of ERK in primary trophoblast cells. Relative mRNA expression levels of GPR54 **(A)** and KP **(B)** in HTR8 cells (white bars) and trophoblast cells (black bars). Protein expression of GPR54 **(C)** and KP-145 and KP-54 **(D)** in HTR8 cells and trophoblast cells. β-actin was used as a loading control. **(E)** HTR8 and trophoblast cells were treated for 10 min with vehicle (-), 1 µM KP antagonist (p356), 100 nM KP or both treatments (KP + p356) in combination in serum-free medium. Phospho- and total ERK1/2 protein levels were determined by Western Blot. **(F)** Quantification of Western Blots for phospho- and total ERK1/2 expression (n = 3). Error bars represent SEM. Statistical significance was tested by ANOVA, columns with different letters represent statistically different values, p<0.05, while same letters indicates no significant difference, p>0.05.

Expression of GPR54 (Fig. 2A) and KP (Fig. 2B) mRNA in primary trophoblast cells and in HTR8 was determined by qPCR. HTR8 was included as a control, being an immortalized trophoblast cell line that has previously been used to study GPR54 function [7]. Expression of GPR54 and KP mRNA was 15.8-fold and 75.5-fold higher in the primary trophoblast cells than in HTR8 cells, respectively. Protein expression of GPR54 and KP was determined by Western Blot (Fig. 2C and D), and consistent with the high level of GPR54 and KP mRNA, higher levels of GPR54 and KP proteins (KP-145 and KP-54) were observed in the primary trophoblast cells in comparison to the level of GPR54 and KP proteins in HTR8 cells which were extremely low.

KP has been shown to activate ERK signalling in GPR54-transfected CHO cells and in the immortalized trophoblast cell line HTR8SVneo [7], [14]. Treatment of HTR8 cells with KP for 10 min, resulted in a mild (1.7 fold) increase in phosphorylated ERK1/2, however, this increase was not statistically significant, p = 0.127 (Fig. 2E and F, left side). In contrast, treatment of primary trophoblast cells with KP for 10 min, resulted in a 3 fold increase in phosphorylated ERK1/2 that was statistically significant, p = 0.0011 (Fig. 2E and 2F, right side, dark grey bar). Co-treatment of the trophoblast cells with KP and p356, a KP antagonist, almost completely blocked KP-induced ERK1/2 phosphorylation and the observed level of ERK1/2 phosphorylation was not statistically different than that of unstimulated cells, p = 0.0828 (Fig. 2E and 2F, right side, black and white bars), while treatment with the KP antagonist (p356) alone had no significant effect of ERK1/2 phosphorylation, p = 0.2947.

### KP inhibits trophoblast migration

**Figure 3:**
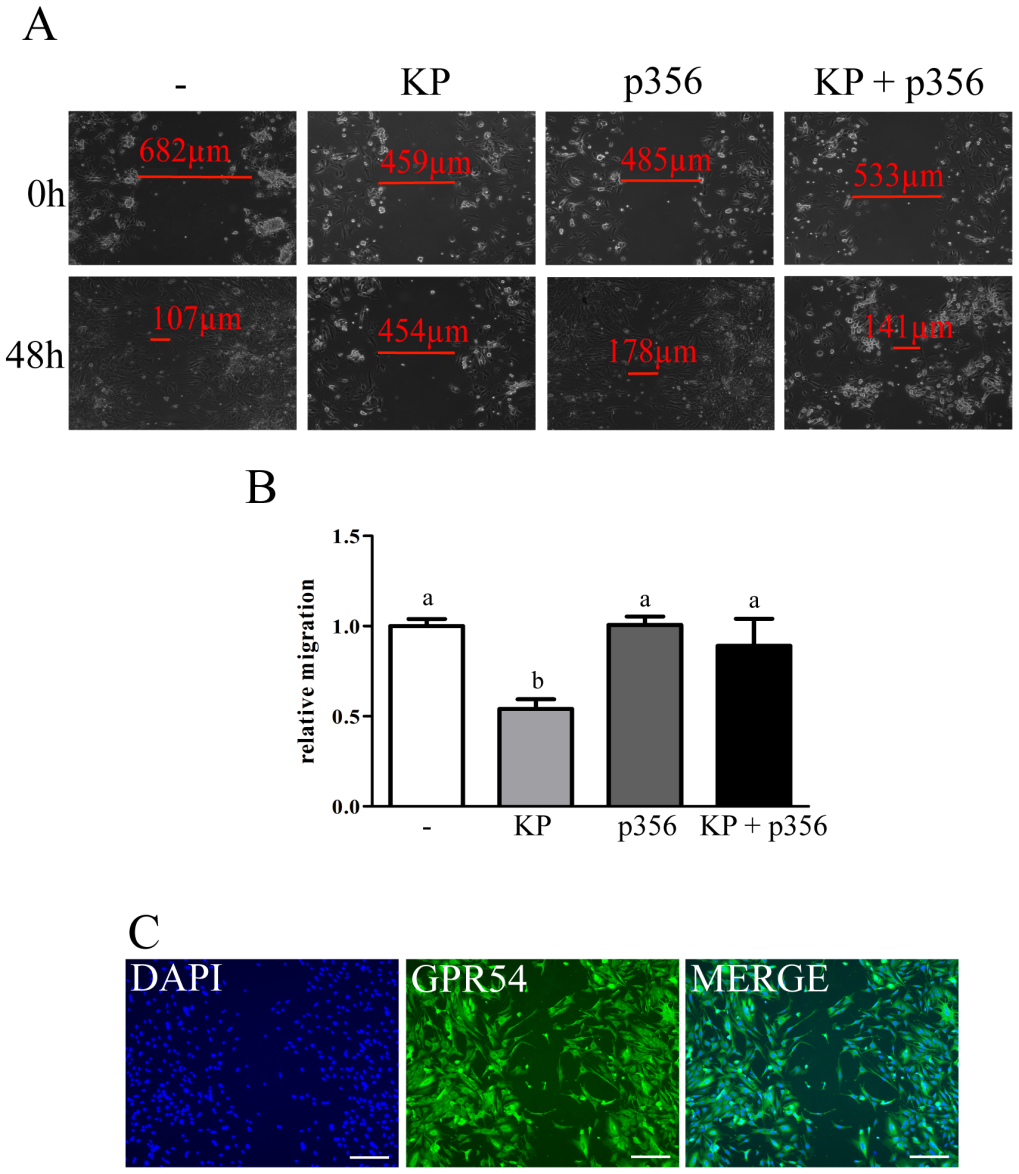
KP inhibits trophoblast migration. **(A)** Images of the scratch migration assay performed on trophoblast cells treated for 48(-), 100 nM KP, 1 µM KP antagonist (p356) or both treatments (KP + p356) in combination. Images were taken immediately after performing the scratch (0 h) and 48 hours later (48 h). **(B)** Quantification of the relative migration of untreated trophoblast cells (white bar) and trophoblast cells treated with KP (light grey bar), p356 (dark grey bar) or KP + p356 (black bar) (n = 6). ANOVA test p<0.05, columns with different letters represent statistically different values, while same letters indicates no significant difference. **(C)** Staining of the migrated cells with DAPI (blue), anti-GPR54 (green) and the merge of staining. Scale bar indicates 200 µm.

Scratch-migration assays were used to determine the effect of KP on trophoblast migration (Fig. 3A). Treatment of trophoblast cells with KP for 48 h inhibited cell migration by 46%, relative to untreated cells (Fig. 3B, white and light grey bars). Co-incubation of trophoblast cells with KP and p356 blocked KP-mediated inhibition of trophoblast migration, restoring the migration to control levels (Fig. 3B, black bar). Staining of the cells that migrated into the scratched area with anti-GPR54 demonstrated that the migrated cells express GPR54 (Figure 3C) and therefore, can be regulated by KP.

### KP regulates the expression of genes involved in cell invasion and angiogenesis

**Figure 4:**
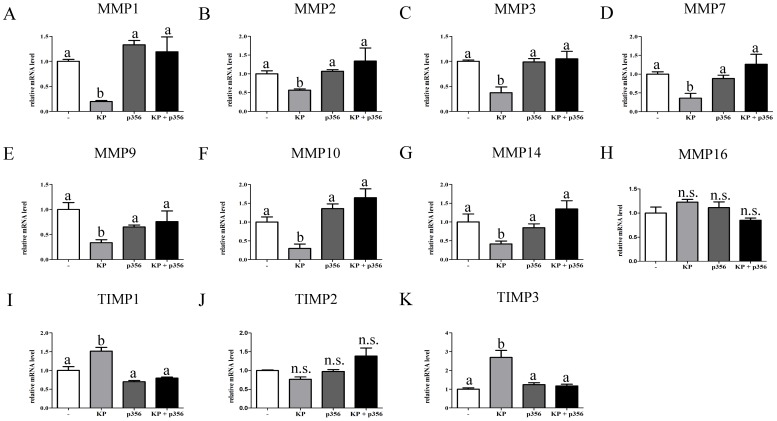
KP supresses expression of MMPs and induces expression of TIMPs. Trophoblast cells were either not treated (white bars) or treated with 100 nM KP (light grey bars), 1 µM KP antagonist (p356) (dark grey bars) or both treatments in combination (black bars) for 48 h. RNA was extracted and expression of MMP1 **(A)**, MMP2 **(B)**, MMP3 **(C)**, MMP7 **(D)**, MMP9 **(E)**, MMP10 **(F)**, MMP14 **(G)**, MMP16 **(H)**, TIMP1 **(I)**, TIMP2 **(J)** and TIMP3 **(K)** was analyzed by qPCR. Error bars represent SEM. ANOVA test p<0.05, columns with different letters represent statistically different values, n.s.  =  no significant difference.

A possible mechanism for KP in regulating the invasion capacity of trophoblast cells is to regulate the expression of genes involved in remodelling the extracellular matrix and angiogenesis. In order to test this hypothesis, a gene array for extracellular matrix genes and adhesion molecules was performed on untreated and treated (with KP for 48 h) trophoblast cells and the expression level of the genes in the array was compared. A list of the genes up and downregulated more than 1.5-fold is shown in Tables 2 and 3, respectively. The Array showed that MMPs 1, 3, 7, 10, 14 and 16 were down regulated while TIMPs 1 and 3 were upregulated. We then examined by qPCR the gene expression of members of the MMP and TIMP families in trophoblast cells. The cells were either not treated or treated with KP or p356 alone or together for 48 h. Figure 4 shows expression of MMP1, 2, 3, 7, 9, 10, 14 and 16, and TIMP1, 2 and 3. All of the MMPs, except for MMP16 were downregulated by KP treatment. Furthermore, co-incubation of KP with the antagonist p356 blocked KP-mediated downregulation (Figs. 4A to H, black bars). On the other hand, TIMP 1 and 3 were upregulated by KP treatment, while co-incubation of KP with the antagonist p356 blocked this upregulation (Figs. 4I and K, light grey and black bars). MMP16 and TIMP2 (Figs. 4H and J) were not significantly regulated by KP treatment.

**Table 2:** List of genes upregulated in the Human Extracellular Matrix and Adhesion Molecules gene array.

Gene name	Gene symbol	Fold change
ADAM metallopeptidase with thrombospondin type 1 motif, 1	ADAMTS1	2,48
catenin (cadherin-associated protein), alpha 1, 102 kDa	CTNNA1	1,61
catenin (cadherin-associated protein), beta 1, 88 kDa	CTNNB1	1,66
catenin (cadherin-associated protein), delta 1	CTNND1	3,08
collagen, type XIV, alpha 1	COL14A1	8,70
collagen, type XV, alpha 1	COL15A1	2,50
fibronectin 1	FN1	1,72
integrin, alpha 1	ITGA1	5,70
integrin, alpha 4 (antigen CD49D, alpha 4 subunit of VLA-4 receptor)	ITGA4	3,32
integrin, alpha V (vitronectin receptor, alpha polypeptide, antigen CD51)	ITGAV	2,38
Kallmann syndrome 1 sequence	KAL1	2,48
phosphoglycerate kinase 1	PGK1	2,85
secreted protein, acidic, cysteine-rich (osteonectin)	SPARC	2,12
TIMP metallopeptidase inhibitor 1	TIMP1	2,22
TIMP metallopeptidase inhibitor 3	TIMP3	1,56
transforming growth factor, beta-induced, 68 kDa	TGFBI	3,06
vascular cell adhesion molecule 1	VCAM1	4,17
versican	VCAN	1,70

**Table 3:** List of genes downregulated in the Human Extracellular Matrix and Adhesion Molecules gene array.

Gene name	Gene symbol	Fold change
collagen, type XII, alpha 1	COL12A1	1,75
connective tissue growth factor	CTGF	1,83
contactin 1	CNTN1	2,19
C-type lectin domain family 3, member B	CLEC3B	4,53
integrin, alpha 2 (CD49B, alpha 2 subunit of VLA-2 receptor)	ITGA2	2,25
integrin, alpha L (antigen CD11A (p180), lymphocyte function-associated antigen 1; alpha polypeptide)	ITGAL	3,09
integrin, beta 1 (fibronectin receptor, beta polypeptide, antigen CD29 includes MDF2, MSK12)	ITGB1	2,04
integrin, beta 2 (complement component 3 receptor 3 and 4 subunit)	ITGB2	1,63
integrin, beta 3 (platelet glycoprotein IIIa, antigen CD61)	ITGB3	2,28
integrin, beta 4	ITGB4	1,97
integrin, beta 5	ITGB5	3,34
laminin, alpha 1	LAMA1	4,25
laminin, alpha 3	LAMA3	1,50
laminin, beta 1	LAMB1	1,65
laminin, beta 3	LAMB3	2,51
matrix metallopeptidase 1 (interstitial collagenase)	MMP1	3,55
matrix metallopeptidase 3 (stromelysin 1, progelatinase)	MMP3	2,95
matrix metallopeptidase 7 (matrilysin, uterine)	MMP7	1,68
matrix metallopeptidase 10 (stromelysin 2)	MMP10	1,81
matrix metallopeptidase 14 (membrane-inserted)	MMP14	1,68
matrix metallopeptidase 16 (membrane-inserted)	MMP16	4,00
neural cell adhesion molecule 1	NCAM1	3,31
platelet/endothelial cell adhesion molecule	PECAM1	2,22
tenascin C	TNC	5,35
ubiquitin C	UBC	14,51

**Figure 5:**
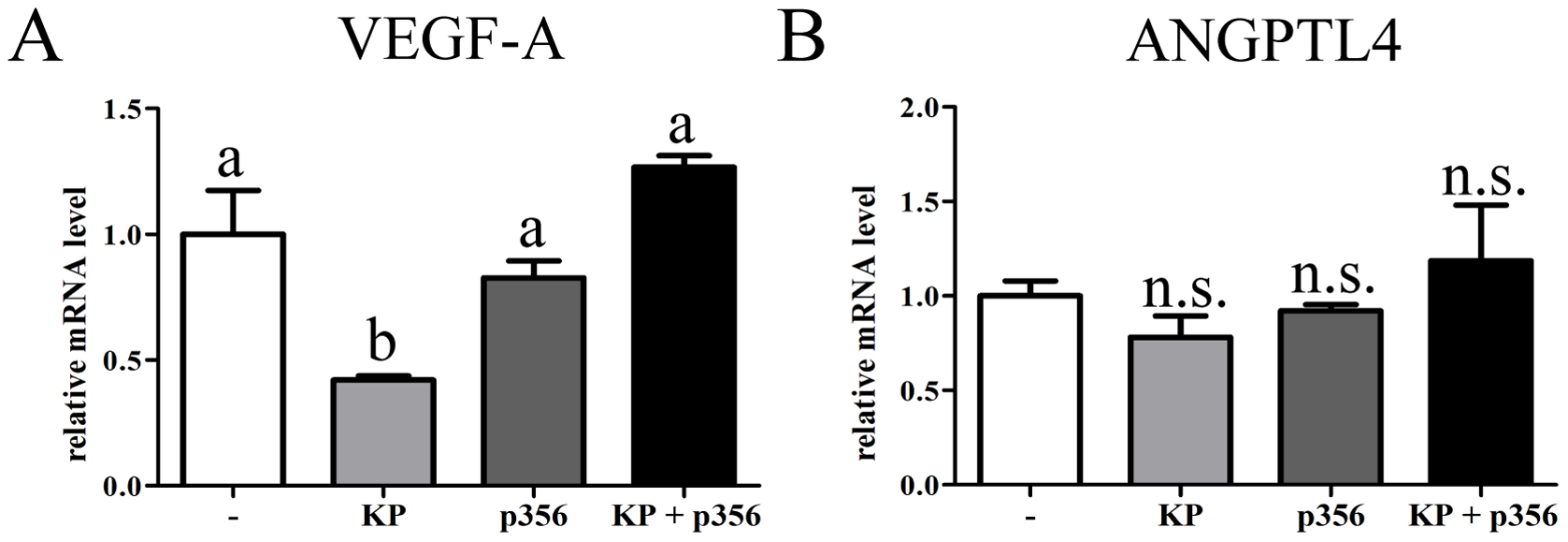
KP regulates expression of VEGF-A, but not of ANGPTL4. Trophoblast cells were either not treated (white bars) or treated with 100 nM KP (light grey bars), 1 µM KP antagonist (p356) (dark grey bars) or both treatments in combination (black bars) for 48 h. RNA was extracted and expression of VEGF-A **(A)** and ANGPTL4 **(B)** was analyzed by qPCR. Error bars represent SEM. ANOVA test p<0.05, columns with different letters represent statistically different values, n.s.  =  no significant difference.

Expression of angiogenic genes was subsequently determined. Figure 5 shows expression of vascular endothelium growth factor A (VEGF-A) and angiopoietin-like protein 4 (ANGPTL4). VEGF-A was downregulated by KP treatment, while co-incubation of KP with the antagonist p356 blocked this downregulation (Fig. 5A, light grey and black bars). However, ANGPTL4 (Fig. 5B) was not significantly regulated by KP treatment.

## Discussion

This study demonstrates that KP inhibits trophoblast migration capacity in primary cultures of first trimester human trophoblast cells and suggesting that KP has a role as one of the inhibitors of EVT invasion during early placental development. This study also shows that GPR54 and KP proteins and transcripts are expressed in trophoblast cells of first trimester human placenta. In addition, this study shows that KP activation of GPR54 stimulates ERK1/2 phosphorylation, demonstrating that the GPR54 receptor is functional in trophoblast cells. Furthermore, KP treatment can inhibit the invasion capacity of trophoblast cells by inhibiting cell migration and by regulating tissue remodelling molecules of the matrix metalloproteinase family (MMPs and TIMPs) and the angiogenic factor VEGF-A.

The expression of GPR54 and KP mRNA and proteins in primary trophoblast cells were much higher than in the HTR8 immortalized trophoblast cell line. Consistent with that the activation of ERK1/2 in trophoblast cells was 3 fold, while the activation of ERK1/2 in HTR8 cells was very weak (1.7 fold) and was not statistically significant. This result is in contrast to the reported activation of ERK1/2 in HTR8 cells [7], however in that report the activation of ERK1/2 varied and in one experiment it was only about 1.7 fold which is weak and similar to the activation we observed in this study. The more robust activation of ERK1/2 in response to KP in trophoblast cells validates the KP-GPR54 signalling pathway in these cells.

MMPs function in the extracellular environment of cells and degrade both matrix and non-matrix proteins. They play central roles in morphogenesis, wound healing and tissue repair. Their activities are regulated by TIMPs [25]. Several studies have stressed the role played by MMPs in extravillous trophoblast invasion and remodelling of spiral arteries [1]. When MMP9 expression is reduced in trophoblast cells in vitro, these cells show a reduced invasion capacity [26]. Their importance in proper invasion of trophoblast cells is highlighted by the observation that MMP1, 3 and 7 are downregulated in EVTs of patients with preeclampsia and intrauterine growth restriction [27], [28]. It has been shown that KP reduces the proteolytic activity of MMP2 in trophoblast explants and primary cultures [11]. In addition, recently, it was shown that KP downregulates the transcription of MMP2 and 9 in HTR8SVneo cells [7]. Here we have shown that KP downregulates the transcription of MMPs 1, 2, 3, 7, 9, 10 and 14 and upregulates the transcription of TIMP1, and 3. This demonstrates a dual regulation of MMPs activity by KP. KP can regulate the MMPs directly by down regulating their transcription and indirectly by upregulating TIMP1 and 3 transcription.

In our study the pattern of regulation of the MMPs and TIMPs genes observed in the gene array experiment for extracellular matrix genes and adhesion molecules (Tables 2 and 3) were confirmed in the qPCR experiments (Fig. 4), except for the down regulation of MMP16 which was not confirmed. Our results show that all of the MMPs and TIMPs examined, except for MMP16 and TIMP2, are regulated by KP treatment. Different regulation of MMP16 and TIMP2 relative to their other respective family has not been documented. The lack of regulation of MMP16 and TIMP2 by KP may indicate that either they do not have a major role in the EVT invasion capacity or that they play an important homeostatic role in trophoblast invasion and should not be negatively regulated by KP.

EVTs also rely on expression of pro-angiogenic molecules in order to invade the decidualized endometrium and transform the maternal spiral arteries into vessels of low resistance, by replacing endothelial cells and vascular smooth cells [1]. We have shown that VEGF-A expression is downregulated by KP. VEGF-A secreted by the trophoblast cells promotes vascularization of the placenta during the first trimester [29]. This result suggests that KP through its down regulation of VEGF-A transcript could negatively affect angiogenesis. We did not observe any changes on ANGPTL4 expression, although this protein has been described as a potent inhibitor of angiogenesis and invasion [30].

In addition, to KP role in down regulating MMP activity and angiogenesis, we have also shown that KP is an inhibitor of migration in primary cultures of trophoblast cells. These results suggest that KP acts at multiples levels to inhibit cell invasion. This action may prove advantageous for restricting the extent of trophoblast invasion during placentation. The active pathways during placentation are finely balanced between positive and negative regulators to ensure the correct level of trophoblast invasion and remodelling of the maternal spiral arteries. Apart from the already mentioned MMP system, Wnt signalling has also been shown to increase trophoblast invasion and may be the regulator responsible for promoting MMP2 activation [31]. Another positive regulator of trophoblast invasion is the Leukemia Inhibitory Factor (LIF), which is able to increase trophoblast invasion by down-regulating integrin-β4 and therefore enhancing cell attachment to extracellular matrix components [32]. β-catenin has recently been shown to regulate trophoblast migration in HTR8 cells [7]. TGFβ1 negatively regulates EVT invasion via multiple mechanisms that include downregulation of plasminogen activator of urokinase (PLAU) and upregulation of TIMP1 and TIMP2 [9], [33]. All these positive and negative regulatory pathways must work in concert and be coordinated in order to achieve proper trophoblast invasion and successful placentation.

In this study, data from 48 h of KP treatment have been presented, but KP was also found to regulate a few of the studied genes after 8 h (data not shown). Gene regulation is a dynamic process and further exploration is required to elucidate the kinetics of KP regulation of the invasion-related genes. It would also be of interest to identify which intracellular signalling pathways are activated by KP and regulate migration in primary trophoblast cells. Similar experiments have recently been performed by Roseweir et al [7] using HTR8 cells. The high level of GPR54 makes primary trophoblast cells a better system to study trophoblast invasion than HTR8. Bilban et al have recently shown how different the gene signatures in EVTs and some immortalized trophoblast cell lines are, therefore underlining how important it is to work with trophoblast primary cultures and verifying the crucial experiments in primary cultures- and subsequently, choosing the right immortalized trophoblast cell line [34]. Based on their findings, choriocarcinoma-derived cell lines (e.g. BeWo, JEG-3 and ACH-3P) could be preferentially used for studies on cell motility and invasion, rather than SV40 large T antigen-selected cell lines (e.g. HTR8 and SGHPL-5). Prospective studies could focus on searching for pregnancy-related conditions in knockout mice for GPR54 and KP genes. KISS1- and GPR54- null mice phenotype have already been described [35], [36], but the physiology and gene expression of the placenta of these mutant fetuses was not tested.

In conclusion, this study demonstrates that human primary trophoblast cells of first trimester express high levels of GPR54 receptor protein and KP peptides and are a suitable model system for studying KP-GPR54 signalling and activity. Our results suggest that KP inhibits trophoblast cells invasion at multiple levels. KP directly inhibts trophoblast cell migration, it downregulates MMP transcription and upregulates TIMP transcription as well as downregulates VEGF-A transcription. This study with primary cultures unravelled novel targets of KP and sets the scene for further studies into the molecular mechanism underlying KP regulation of trophoblast cell invasion.
